# Modeling Genome-Wide Dynamic Regulatory Network in Mouse Lungs with Influenza Infection Using High-Dimensional Ordinary Differential Equations

**DOI:** 10.1371/journal.pone.0095276

**Published:** 2014-05-06

**Authors:** Shuang Wu, Zhi-Ping Liu, Xing Qiu, Hulin Wu

**Affiliations:** Department of Biostatistics and Computational Biology, University of Rochester, Rochester, New York, United States of America; Leibniz-Institute for Farm Animal Biology (FBN), Germany

## Abstract

The immune response to viral infection is regulated by an intricate network of many genes and their products. The reverse engineering of gene regulatory networks (GRNs) using mathematical models from time course gene expression data collected after influenza infection is key to our understanding of the mechanisms involved in controlling influenza infection within a host. A five-step pipeline: detection of temporally differentially expressed genes, clustering genes into co-expressed modules, identification of network structure, parameter estimate refinement, and functional enrichment analysis, is developed for reconstructing high-dimensional dynamic GRNs from genome-wide time course gene expression data. Applying the pipeline to the time course gene expression data from influenza-infected mouse lungs, we have identified 20 distinct temporal expression patterns in the differentially expressed genes and constructed a module-based dynamic network using a linear ODE model. Both intra-module and inter-module annotations and regulatory relationships of our inferred network show some interesting findings and are highly consistent with existing knowledge about the immune response in mice after influenza infection. The proposed method is a computationally efficient, data-driven pipeline bridging experimental data, mathematical modeling, and statistical analysis. The application to the influenza infection data elucidates the potentials of our pipeline in providing valuable insights into systematic modeling of complicated biological processes.

## Introduction

Influenza A virus is an important respiratory pathogen that poses a considerable threat to public health each year during seasonal epidemics and even more so when a pandemic strain emerges. The immune response to viral infection is a dynamic process and is regulated by an intricate network of many genes and their products. Understanding the dynamics of this network will shed light on the mechanisms involved in controlling influenza infection within a host and is also important for developing new and effective treatment strategies. Recently, several studies have been performed to monitor the within host genome-wide expression patterns of immune responses over time to influenza infection [1], [2]. Analyzing such time course gene expression data requires the use of advanced statistical and computational approaches developed specifically for time series data instead of the standard methods for the traditional snap-shot or vector expression data. In particular, reverse engineering the gene regulatory networks (GRNs) from the time course expression data using mathematical models, especially dynamic network models, is of increasing research interest. In this paper, we will use a high-dimensional ordinary differential equation (ODE) model to construct the genome-wide dynamic GRN of influenza infected mouse lungs. This model will provide quantitative measures of the global response of the immune system to influenza infection *in vivo* and also help us better understand the virus-mediated immunopathology in a systematic way.

Previously developed computational approaches for inferring GRNs from gene expression data are either not efficient in describing dynamic regulations between genes or restricted to small-scale networks. For example, information theory models [3]–[5] are basically correlation networks. They are simple and easy to compute, but they are static models and do not take into account that multiple genes could co-regulate a target gene. Boolean networks [6]–[9] are discrete dynamic networks in which the state of a gene is represented by a binary variable that is either on or off. Such models are limited because the continuous nature of gene expressions cannot be described adequately by only two states. Bayesian networks (BNs) [10]–[16] make use of the Bayesian rule and provide a flexible framework for combining different types of data and prior knowledge. Time course data can be used to reconstruct dynamic BNs [13], [15], but the optimization of the network usually requires very high computational cost, so the applications are mostly limited to small systems. The vector autoregressive (VAR) and state space models (SSM) models are discrete dynamic models which usually require equally-spaced and intensive time-series data in order to obtain reliable inference results for model parameters [17]–[19]. Differential equation models [20]–[28] quantify the change rate (derivative) of the expression of one gene in the system as a function of expression levels of all related genes. It is a directed network graph model and the dynamic feature of the GRN is automatically and naturally quantified. Moreover, both up and down regulatory relationships between genes as well as self-regulations can be appropriately captured. A major challenge to use differential equation models for reconstructing GRNs is how to identify the model structure and estimate parameters efficiently in high-dimensional models. Excellent reviews on diverse data-driven modeling schemes and related topics can be found in [29]–[31].

Our objective is to develop a computationally efficient method that is feasible to reverse engineer genome-wide dynamic GRNs using high-dimensional ODE models. We propose a novel pipeline to reconstruct dynamic GRNs from time course gene expression data by combining a series of cutting-edge statistical techniques. A major difference of our method from the work by others is that we do not discretize the ODE model by transforming the derivatives into differences. Our approach uses the smoothed estimates of the first derivatives of the gene expression profiles. It has been shown that such smoothed-based method produces better parameter estimates for ODEs than those discrete-time models based on differences, because it is more robust to the noises in data [27], [32]. In addition, we use several advanced statistical techniques such as the two-stage estimation method for ODEs and the penalization-based variable selection method to speed up the identification of the network structure, so that our method is capable of handling large-scale, including genome-wide, gene regulatory networks.

In a typical gene expression experiment, tens of thousands of genes are measured simultaneously, but only a fraction of them are associated with the biological process of interest or a particular stimulus. Since it is reasonable to include only these “responsive” genes in the ODE network model, the first step in the GRN modeling is to identify temporally differentially expressed genes, i.e., genes with expression levels that change significantly over time. Within the set of differentially expressed genes, which usually ranges from several hundreds to thousands, many genes behave similarly during the experimental period, making it difficult to distinguish their expression patterns based on the time course data. We assume that genes with similar expression patterns have similar biological functions under the same experimental conditions and cluster these similarly behaved genes into co-expressed modules [33], [34]. Similar assumptions have been adopted by [4], [35] and [36], [37] have clearly shown in their studies that genes in the same expression cluster were enriched for similar biological functions. We treat these co-expressed modules as the nodes of the GRN, which significantly reduce the dimension of the ODE model and the associated computational cost. Moreover, this approach can help avoid the identifiability issue of the ODE model, as the gene expressions of these modules are sufficiently different from each other [38].

It is well known that biological systems are seldom fully connected and most nodes are only directly connected to a small number of other nodes [39], consequently, the GRN is a sparse network. In the key step of identifying the sparse structure of the network, i.e., identifying the significant edges between modules, we couple the advanced parameter estimation method for ODE models with statistical variable selection techniques to perform model selection. This method avoids numerically solving the differential equations directly and more importantly, it allows us to perform model selection and parameter estimation for one equation at a time, which significantly reduces the computational cost. Once the network structure is determined, we can refine the estimates of the model parameters using more refined optimization methods, and then annotate biological implications based on the refined network through the functional enrichment analysis.

## Results and Discussion

### Pipeline of reverse engineering genome-wide GRN

**Figure 1:**
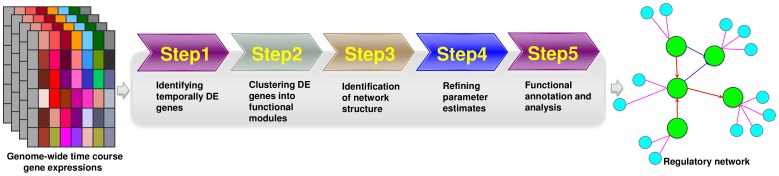
The road map of the proposed pipeline for reconstructing genome-wide dynamic GRNs.

The road map of the proposed pipeline is summarized in Figure 1. Our pipeline enables the global analysis of genome-wide time course gene expression data and outputs the dynamic GRN. The pipeline consists of five steps: (i) detection of temporally differentially expressed (DE) genes; (ii) clustering differential genes into co-expressed modules; (iii) identification of network structure; (iv) parameter estimate refinement; and (v) functional enrichment analysis. A series of advanced statistical techniques are employed, including the functional principal component analysis (FPCA) with hypothesis testing, time course gene expression clustering, nonparametric mixed-effects modeling, and parameter estimation and statistical inference for ODE models. The technical details of our pipeline are described in the Methods section. D-NetWeaver, a graphical user interface (GUI) software, implements this pipeline and is available at https://cbim.urmc.rochester.edu/software/d-netweaver/.

### Experimental data

We apply the proposed reverse engineering pipeline to study the genome-wide regulatory interactions of the dynamic GRN in mouse lungs after perturbation of the immune system by influenza infection. The time course microarray gene expression data were collected by Pommerenke *et al.* [2] in mouse lungs infected with influenza A virus PR8 (H1N1). The genome-wide transcriptome patterns were measured at days 1, 2, 3, 5, 8, 10, 14, 18, 22, 26, 30, 40 and 60 post infection (p.i.). Three mice were prepared as independent biological replicates at each day p.i., except for day 3 and 5, where there were 6 replicates. Nine mice were mock-infected and their gene expression data were collected as baseline measurements (day 0). The total number of genes in the data set is 27527.

### Global temporal variation of differentially expressed genes

**Figure 2:**
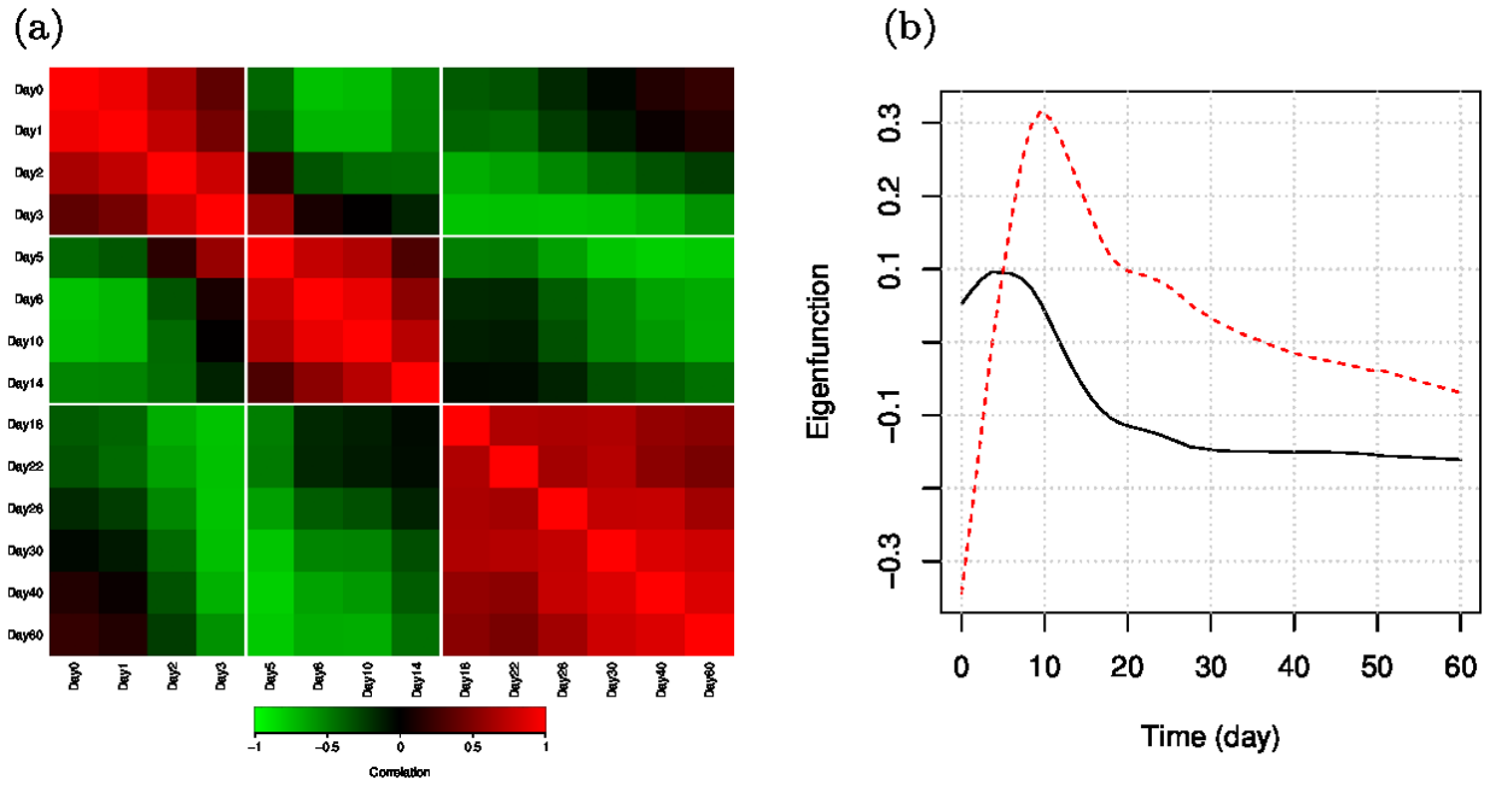
Overview of the temporal variations of DE genes. (a) Correlation matrix between every pair of time points indicates three major transcriptional phases. (b) Estimates for the first two eigenfunctions (first-black solid, second-red dashed) for the DE genes.

We identify 3666 genes with temporally differential expression patterns after viral infection at the false discovery rate (FDR) of 0.01. From the pair-wise correlation matrix of these DE genes (Figure 2 (a)), we can clearly see that the temporal transcriptional variations can be partitioned into three phases: early (up to day 4 p.i.), intermediate (days 5–17 p.i.) and late (days 18–60 p.i.). These three transcriptional phases after influenza infection are likely to be associated with the major phases of the immune response: the innate, the adaptive, and the memory immune response, as well as the tissue repair process. In addition, a functional principal component analysis [40] of the DE genes reveals that the majority of temporal variations in these genes are reflected by two orthogonal representative patterns, which we refer to as the eigenfunctions (Figure 2 (b)). The gene expression profile of each gene can be represented as a linear combination of these two eigenfunctions. The first eigenfunction (explaining 79.7% of the total variation) has an early peak around day 4 p.i. and gradually drops until around day 18 p.i., followed by a sustained slow decrease until day 60 p.i. The second eigenfunction (explaining 18.2% of the total variation) exhibits a rapid increase in the beginning, reaching a peak at around day 10 p.i., and then decreases afterwards. From the shapes of these two eigenfunctions, we can see that the temporal variations in the DE genes mostly occur around day 4 and day 10 p.i., which are likely to be associated with the innate immune response and T cell response, respectively. We also notice that genes with large negative loadings on the first eigenfunction are activated after day 10 p.i. and increase till day 60 p.i. These late-activated genes probably correspond to the infiltration of B cells into the lungs and the formation of bronchus-associated lymphoid tissue (BALT) and tissue repair in the lung.

### Identification of distinct temporal expression patterns

**Figure 3:**
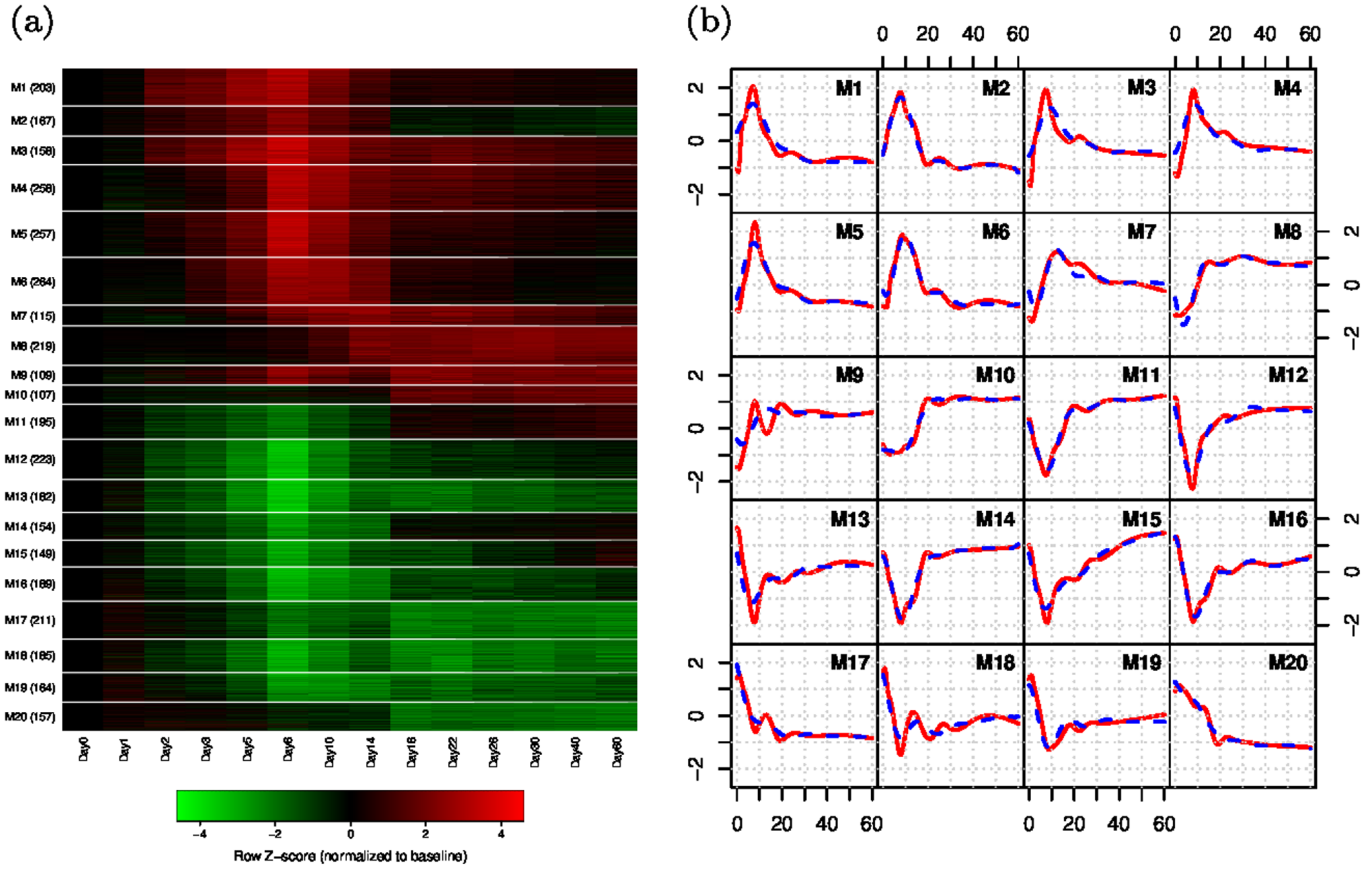
Temporal expression profiles of DE genes. (a) The DE genes are clustered into 20 modules (M1–M20) and the number of genes in each module is displayed in the parentheses. Shown are standardized gene expressions normalized to day 0. (b) The smoothed mean expression curve obtained from (6) (red solid) for each module overlaid with the refined estimate from the linear ODE model (blue dashed).

To view the temporal gene expression patterns after virus infection on a more refined scale and also to facilitate the following network modeling, we cluster the DE genes into co-expressed modules. A total of 20 modules are obtained (Figure 3, Table S1). Treating the gene expression profiles within the same module as longitudinal replicates, we obtain the smoothed mean expression curve and the corresponding first order derivative for each module using model (6) in the Methods Section. Please note that Figure 3(b) is plotted in the real time scale. It may be difficult to see the differences in the module patterns, because most of the temporal variation occurred in the first few days. The heat map of the standardized gene expression profiles for each module in Figure 3(a) better shows the pattern differences. Functional enrichment analysis is carried out using DAVID [41]. Due to space limitation, we only display some selective functional annotations in Table 1. We can see that several modules have functions related to the immune response, such as innate immune response, positive regulation of adaptive immune response, T cell activation, B cell receptor signaling pathway. More details of the enriched functions for each module and the genes associated with each functional term can be found in Table S2.

**Table 1:** The inward and outward regulations in the module-based regulatory network.

Module	Inward Influence	Outward Influence	Functional Annotation
M1	1, 4^−^, 6^−^, 7^−^	1, 3^−^, 5, 6^−^, 7^−^, 9^−^, 12^−^, 13^−^, 14, 15, 17^−^, 18	Innate immune response, antigen processing and presentation of exogenous peptide antigen via MHC class I, cytokine-cytokine receptor interaction, NK cell mediated cytotoxicity, cytokine mediated signaling pathway
M2	3^−^, 4, 7, 12^−^, 14^−^, 17		Immune response, apoptosis
M3	1^−^, 4, 6, 7^−^	2^−^, 4, 5^−^, 11, 16, 19	Defense response, leukocyte and lymphocyte activation
M4	3, 5, 7^−^, 18	1^−^, 2, 3, 5, 7^−^, 11^−^, 12^−^	Activation and differentiation of lymphocyte and leukocyte, T cell activation, hemopoietic or lymphoid organ development, T helper cell surface molecules, T cytotoxic cell surface molecules
M5	1, 3^−^, 4, 5, 6^−^	4, 5, 16^−^	Activation and proliferation of T cell and lymphocyte, regulation of cytokine production
M6	1^−^, 6^−^, 7^−^, 12^−^, 15^−^, 19^−^	1^−^, 3, 5^−^, 6^−^, 8^−^, 12, 14, 19	M phase, mitotic cell cycle, cell division
M7	1^−^, 4^−^, 8^−^, 9^−^, 15^−^, 19^−^	1^−^, 2, 3^−^, 4^−^, 6^−^, 8^−^, 10, 12, 16^−^, 18	B cell activation, B cell receptor signaling pathway, antigen processing and presentation of exogenous peptide antigen via MHC class II, intestinal immune network for IgA production
M8	6^−^, 7^−^, 12^−^, 19^−^	7^−^, 9^−^, 10^−^, 13, 15, 17, 18, 19, 20	Epidermis development, primary immunodeficiency, epithelial cell differentiation, epithelium development
M9	1^−^, 8^−^, 15^−^, 19^−^	7^−^, 16^−^, 19, 20^−^	Epithelium development
M10	7, 8^−^, 14^−^, 15^−^, 19, 20^−^		Regulation of RNA metabolic process, positive regulation of epithelial cell differentiation
M11	3, 4^−^		Transmembrane receptor protein tyrosine kinase signaling pathway
M12	1^−^, 4^−^, 6, 7, 19^−^	2^−^, 6^−^, 8^−^, 14	inositol phosphate metabolism
M13	1^−^, 8, 19^−^	18, 19	Drug metabolism, negative regulation of cell migration
M14	1, 6, 12, 15, 20^−^	2^−^, 10^−^, 20	vasculature development
M15	1, 8, 19^−^	6^−^, 7^−^, 9^−^, 10^−^, 14	Microtubule-based process, ciliary or flagellar motility
M16	3, 5^−^, 7^−^, 9^−^		negative regulation of cellular component organization
M17	1^−^, 8, 19^−^	2	ECM-receptor interaction
M18	1, 7, 8, 13, 18^−^, 19^−^	4, 18^−^, 19^−^	Tight junction
M19	3, 6, 8, 9, 13, 18^−^	6^−^, 7^−^, 8^−^, 9^−^, 10, 12^−^, 13^−^, 15^−^, 17^−^, 18^−^, 20^−^	Drug metabolism
M20	8, 9^−^, 14, 19^−^	10^−^, 14^−^	Negative regulation of molecular function

The negative sign indicates a negative coefficient in the linear ODE model; otherwise the coefficient is positive. The underlined modules are hub modules with the most outward regulations.

The innate immune response is the first line of defense against pathogens and acts more fast in comparison to the adaptive immune response. We can see that genes in M1 respond to the viral infection immediately with expression levels rapidly increasing up to around day 8 p.i. and then quickly dropping back to baseline levels after day 14 p.i. (Figure 3(a)). Consistent with the early activated expression patterns, we find that genes in M1 are mostly related to the innate immune response (Table 1 and Table S2). For example, *Tnf* is one of the most important proinflammatory and proimmune cytokines and is known to be critically involved in the regulation of infectious and inflammatory phenomena [42]. Another important cytokine gene, *Infg*, is also found in M1. Interferon gamma (IFN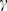), which is encoded by *Ifng*, is often known as the macrophage-activating factor. It has the ability to inhibit viral replication directly and thus is crucial for innate immunity against viral infections [43]. The chemokine receptors *Ccr2* and *Ccr5* play important roles in the recruitment of monocytes/macrophages and T cells. *Ccr2* and *Ccr5* knockout mice appear to have impaired macrophage function and dendritic cell activation in the lung, which in turn considerably alter natural killer (NK) cell function and innate immunity in mice [44], [45].

Genes in M2 and M3 are also activated in the early phase, but their gene expression patterns differ from those of M1 after day 14 p.i. As shown in Figure 3(a), gene expressions of M2 stabilize to levels lower than baseline after day 14 p.i., while those of M3 remain higher than baseline until day 60 p.i. The biological functions enriched in M2 include DNA replication and apoptosis after viral infection. Some T cell related functions are also found in M2 but they are not significantly enriched (Table 1 and Table S2). M3 is mainly enriched for lymphocyte and leukocyte activations, including the important innate immune modulator *Bcl3*, which is known to be associated with secondary lymphoid organ development, cell survival, and inflammatory cytokine gene expressions, and is able to prevent acute inflammatory lung injury in mice by restraining emergency granulocyte accumulation [46], [47] (Table 1 and Table S2).

The transition to the intermediate phase is marked by the activation and proliferation of T cells and lymphocytes. We can see that both M4 and M5 feature strong augmentations of gene expression levels starting at around day 3 p.i. and peaking around day 8 p.i. While the gene expression levels of M5 drop quickly after day 14 p.i., those of M4 still remain considerably higher than baseline levels till day 60 p.i. (Figure 3(a)). There are several T cell signature genes in M4, such as *Cd28*, *Tcra*, *Cd3d*, *Cd3g* and *Cd3e* (Table S2). *Cd28* is a receptor on T cells and it provides a major costimulatory signal upon binding to target ligands B7-1 and B7-2. Such constimulation via *Cd28* is essential for initiating antigen-specific T cell responses, upregulating cytokine expression and promoting T cell expansion and differentiation [48]. *Cd28*-deficient mice have impaired proliferative responses to antigen and anti-CD3 monoclonal antibody activation, but still display significant cytotoxic responses and delayed-type hypersensitivity after virus infection, suggesting that alternative costimulatory pathways may exist [49]. It has been shown that the costimulatory pathway involving *Cd27* (M5, Table S2) is critical for T cell expansion and survival and also for the induction of long-term memory. Although both *Cd27* and *Cd28* make crucial contributions in the generation of antigen-specific T cells, *Cd27* appears as a major determinant for CD8 T cell priming at the site of infection. In addition, both receptors induce the expansion of virus-specific T cells, but *Cd28* promotes cell cycle entry, whereas *Cd27* enhances the accumulation of newly activated T cells by stimulating cell survival [50].

As shown in Table 1 and Table S2, genes in M6 are mostly associated with cell cycle and division. B cell related functions are enriched in M7, indicating the recruitment of B cells into the lung (Table 1). Accordingly, we find that the gene expression levels of M7 start to increase after around day 5 p.i., reaching a peak at around day 14 p.i., and continue to be highly expressed till day 60 p.i. (Figure 3(a)). Many of the genes in M7 are known to be B cell markers or involved in B cell regulation, such as *Cd19*, *Bank1*, *Blnk*, *Cd79a*, *Cd79b*, *Tnfrsf13b* and *Tnfrsf13c* [2], [51] (Table S2). In addition, M7 is enriched for antigen processing and presentation of exogenous peptide antigen via major histocompatibility complex (MHC) class II, whereas M1 is enriched for antigen processing and presentation of exogenous peptide antigen via MHC class I (Table 1). Exogenous antigens enter the MHC class I pathway of antigen-presenting cells (APCs) by a process called “cross presentation” and this process is thought to be crucial for priming CD8-dependent responses against pathogens. MHC II molecules associate with peptides derived from exogenous antigens internalized by endocytosis and they are essential for CD4 T cell recognition of antigen-presenting cells [52]. The ability of MHC II molecules to process and present major MHC II-restricted antigens is known to be regulated by dendritic cells (DCs) [53].

M8, M9 and M10 share similar gene expression patterns, which gradually increase after viral infection and stay at a stabilized level until day 60 p.i. The difference among these three modules is that gene expression levels in M8 start to increase around day 10 p.i., while those in M9 and M10 start around day 5 and day 18 p.i., respectively (Figure 3(a)). M8 is enriched for the epithelial cell differentiation and development, so this module is likely to be associated with the tissue repair process in mouse lungs. Some genes in M9 and M10 are also related to the epithelial cell differentiation and development, but these functions are not significantly enriched in these two modules (Table S2). M11–M20 have down-regulated gene expression patterns, and interestingly, we find that the enriched functions in these modules are mainly house-keeping biological functions, such as cellular process, development, metabolism and binding. Down-regulation in a cell is the process of decreasing the quantity of a cellular component, which may be a protein or a receptor or RNA, in response to an external signal. Here these down-regulated expression levels may reflect the death of the lung epithelial cells or their impairment due to virus infection.

### Regulatory network between modules

The co-expressed modules can be considered as “super-genes” and the intra-module functional annotations presented in the previous section summarize the biological functions of these super-genes. Besides the intra-module functional annotations, we are also interested in building a functional landscape of the genome-wide regulatory network in response to viral infection by constructing a dynamic network between these modules. Applying the two-stage decoupling approach and the SCAD variable selection technique (see Methods), we are able to identify the structure of the module-based network through the linear ODE model (5). The inferred network is sparse, with 90 regulatory relations between 20 modules and only 3 to 6 inward regulations for each module. Since the identified between-module regulatory relationships depend on appropriately chosen tuning parameters in the SCAD method, we randomly perturbed the selected tuning parameters by up to 20% and observed that the structures of the original network and the networks constructed using the perturbed tuning parameters were very close (results not shown), indicating that our data-driven network is robust to the tuning parameter choices. Conditional on the identified network structure, the regulatory parameter estimates are refined using the nonlinear least squares. The estimated expression curves from the refined ODE model are displayed as dashed lines in Figure 3(b). We can see that these refined curve estimates from the ODE model closely follow the mean expression trend for each module.

**Figure 4:**
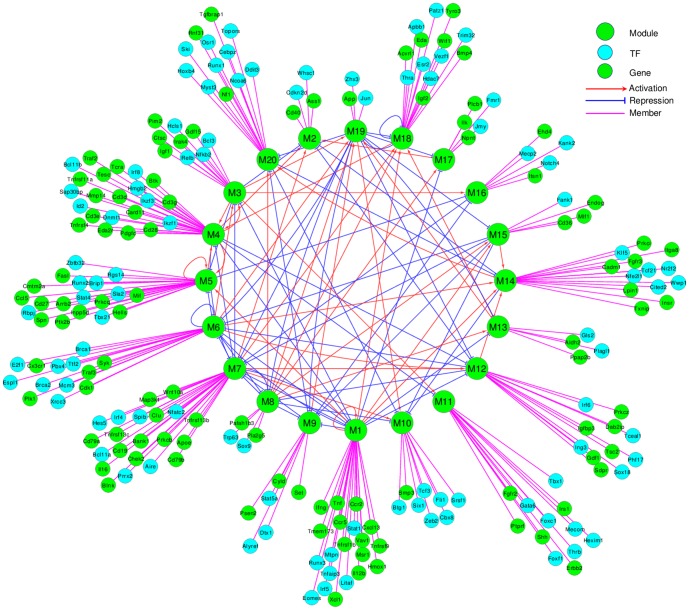
The module-based gene regulatory network constructed by the linear ODE model from the viral infection gene expression data (the inner circle). Selective important regulators and genes identified in each module are shown in the outer circle.

The inferred module-based GRN is visualized in the inner circle of Figure 4 and the detailed information about the inward and outward regulatory relationships between modules is summarized in Table 1. The negative signs in the table correspond to negative coefficients in the refined ODE model. They indicate that the regulatory effects of the other modules on the changing rate of the target module's expression are negative. The positive coefficients in the refined ODE model can be interpreted similarly. We find that Modules 1, 3, 4, 6, 7, 8 and 19 have the most outward regulations, indicating their crucial roles in this network, and we refer to them as the “hub” modules.

Linking the functional annotations and the topology of the gene network identified through the ODE model (Figure 4) can help us understand the functional linkages and associations between these modules, and thus better understand the dynamic regulatory relationships of the whole immune system. Taking M1 as an example, we can see from Table 1 that it regulates most of the other modules, indicating the crucial role of this module in the immune response. M1 is associated with the innate immune response, which provides a generic but immediate defense against pathogens. Since the innate immune response is the first line of defense against invading microbes, it is likely to have influences on many other components or processes in the immune system. For example, it is known that the innate immune response can activate and regulate the antigen-specific adaptive immune response in host defense [54], [55], consistent with M1 regulating M5 and M7, which are enriched as “T cell and lymphocyte activation” and “B cell activation”, respectively (Table 1). For the inward regulations, we find that M1 is regulated by M4, M6, M7 and itself. Many cells are activated during the innate immune response, so cell cycle and division, the main functions of M6, should be involved in the innate immune response. M4 and M7 are both associated with the adaptive immune response, so the regulation of M1 by M4 and M7 indicates that the adaptive immune response has an impact on the innate immune response. This is consistent with the fact that the migration and activities of innate cell types such as neutrophils, monocytes, macrophages, and dendritic cells can be regulated by secretion of cytokines and chemokines by lymphocytes, in particular several T cell subtypes [56], [57]. The self-regulation of the innate immune response has also been discussed in literature. For example, neutrophils have interactions with different components of the innate immune system and can differentially influence the innate immune response [57]. These inter-module regulatory relationships imply the cooperative interactions of these network units and the dynamics of the immune responses after the viral infection.

To better understand the inter-module regulatory relationships on the gene level, we compiled 2389 mouse transcription factors (TFs) from FANTOM [58] and Uniprot [59] (see Table S3) and identified the TFs contained in each module. Some important TFs and genes are shown in the outer circle of Figure 4. Within each module, the TFs can be considered as the functional regulators that mediate the module to perform certain functions, while between modules, the regulatory relationships identified through our data-driven method, may imply potential interactions of the TFs in one module with the TFs and genes in other modules. In other words, if module B regulates module A, the regulators/TFs of a gene in module A are likely to be included in module B.

We find that many of the regulatory interactions identified in our data-driven GRN are already known. For example, *Irf4*, *Irf5* and *Ir8*, belonging to M7, M1 and M4, respectively, are members of the interferon regulatory factor (IRF) family that play versatile and critical roles in the modulation of cell differentiation, development and function of immune cells. It is known that *Irf5* plays an important role in regulating the innate immune response. It activates Type I interferon (IFN) and proinflammatory cytokines and chemokines upon virus infection[60]. It also has a critical role in the regulation of B-cell differentiation [61], which is in line with M1 regulating M7 in our inferred GRN. *Irf4* and *Irf8* are structurally-related and both support T cell differentiation and B cell development [60]. *Irf4* is known to regulate toll-like receptor (TLR) signaling and the transcriptional regulation of proinframmatory cytokine genes such as *Il12b* (M1) [60], [62], consistent with M7 regulating M1 in our inferred network. Both *Irf4* and *Irf8* regulate various macrophage-related genes such as those encoding Cathepsin C (*Ctsc*, M3) and Scavenger receptor (*Msr1*, M1) [60], [63]. In addition, they strongly induce *Irf5* (M1) transcripts, whereas *Irf5* may also mediate the effects of *Irf8* on the innate immune responses in macrophages [63].

### Regulatory relationships within modules

**Figure 5:**
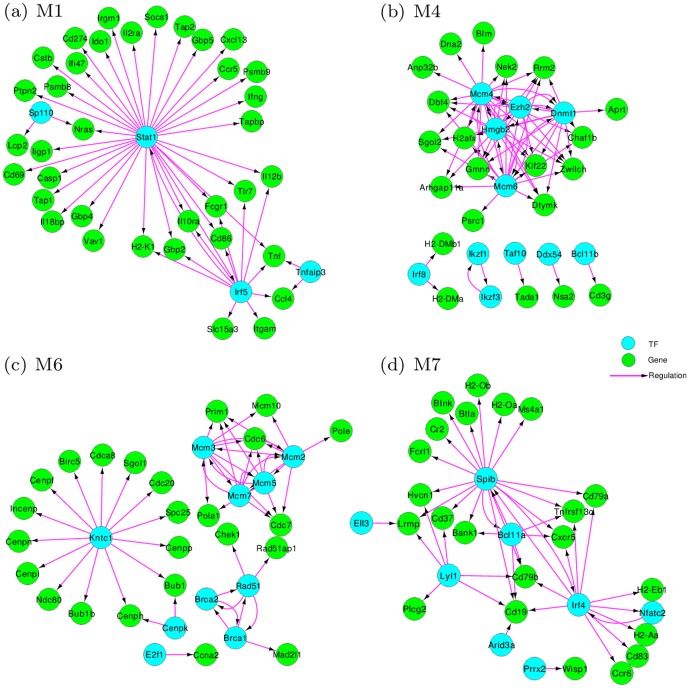
Intra-module regulatory relationships for four modules M1 (a), M4 (b), M6 (c) and M7 (d). TFs are shown in aquamarine and target genes are shown in green. Isolated TFs and genes are not shown.

Our module-based GRN represents the global regulatory relationships between multiple co-expressed gene sets, which is a higher level regulation compared to the TF-gene interactions. Each of the modules assembles the intra-module regulations of many genes to fulfill certain biological functions and interacts with other modules cooperatively after viral infection. In order to decipher the intra-module regulations, we integrate the regulatory linkages between the TFs and target genes, and build the regulatory relationships within each module (see Methods). For illustration, we only show the transcriptional regulatory relationships in four modules M1, M4, M6 and M7 (Figure 5). The detailed list of regulatory relationships of all modules can be found in Table S4.

Many interesting intra-module regulatory relationships have been identified. For instance, it is known that *Irf5* in M1 is a member of the interferon regulatory factor (IRF) family and it regulates the chemokine gene *Ccl4*, whose protein is a chemoattractant for NK cells [59]. The TF *Stat1* (signal transducer and activator of transcription 1) in M1 is involved in the cellular responses to interferons [64]. The regulatory relationships of *Stat1* with its targets *Ifng*, *Gbp2*, *Gbp4*, *Il12a* and *Tap1* indicate the responsive regulations after viral infection, i.e., the activation of the innate immune response and the activated functions of NK cells and cytokine-cytokine receptor interactions, which are consistent with the functional annotation for M1 shown in Table 1.

In M4, the gene regulations between the IRF family member *Irf8* and its targets *H2-DMa* and *H2-DMb1* play critical roles in the immune response [51]. The gene regulations between lymphoid transcription factors *Ikzf1* and *Ikzf3* imply their mediation of hematopoietic cell differentiation and T cell development [65]. We also find that TF *Bcl11b*, which is known as a tumor-suppressor protein involved in T-cell lymphomas, regulates *Cd3g*, a T cell receptor CD3 complex gene [59]. Moreover, the cell-cycle related regulators *Mcm4* and *Mcm6* are found to be highly connected with their targets. These T-cell and cell-cycle related intra-module regulatory relationships enrich the module function of activation and proliferation of T cells. From the inter-module perspective, M4 regulates M1, indicating the cooperative influences of innate immune responses and adaptive immune responses of T cell activation and proliferation after influenza A virus infection.

The biological functions most significantly enriched in M6 and M7 are “cell cycle and division” and “B cell differentiation”, respectively. Accordingly, we find that in M6, the interactions between the TF *Kntc1* and its targets *Cdca8*, *Cdc20* and *Cdc25c* are closely related to cell cycle and cell division. In addition, TFs *Brca1* and *Brca2* are well known as the major mediators of DNA damage in breast cancer research and the TF *Mcm3* is crucial for DNA replication and cell proliferation [59]. The gene regulations between these TFs and their targets *Rad51*, *Rad51ap1*, *Mcm10* and *Pola1* delineate the responses of DNA replication in cell division and DNA repair after viral infection. In M7, the regulation between *Bcl11a* and *Tnfrsf13b* plays a crucial role in the stimulation of B cell function and the regulation of humoral immunity [59]. The gene regulations between TFs *Irf4*, *Spib* and their target genes *Cd19*, *Cd79a*, *Cd79b*, *Cd37* and *Cd83* indicate the B cell proliferation and differentiation in response to influenza infection. They assemble the antigen receptors of B-lymphocytes in order to decrease the threshold for antigen receptor-dependent stimulation [66]. The interferon regulatory factor *Irf4* regulates the major histocompatibility complex genes *H2-Aa* and *H2-Eb1*, which indicates the control of B-cell proliferation and differentiation. These intra-module regulations demonstrate the detailed regulatory relationships of these interconnected modules respectively.

Besides the four modules above, we also find some interesting intra-module regulatory relationships in other modules. For example, M8 is associated with epithelial cell differentiation and development, which reflects the redevelopment or repair of the immune system after viral infection. The TF *Trp63* in M8 is considered as a master regulator of stratified epithelial development [67] and the regulatory relationship between *Trp63* and gene *Krt14* illustrates the underlying transcriptional program during the repair process. In addition, there are TFs and genes in each module whose regulatory interactions are currently not documented in the literature. For simplicity of presentation, these isolated TFs and genes are not shown in Figure 5. Although not connected in the current knowledge-based intra-module regulatory linkages, many of the isolated TFs and genes in the same module are found to be related to the same biological functions or processes. For instance, in M4, the TFs *Ezh2*, *Tox* and TNF-receptor-associated factor (TRAF) family members *Traf2*, *Tnfrsf4*, *Tnfrsf11a*, *Tradd* and *Tnfrsf18* are known to be associated with the activation of NF-kappaB signaling pathway and the critical roles in CD4+ T cell responses as well as in T cell-dependent B cell proliferation and differentiation after influenza A virus infection [51]. It is possible that there are undiscovered regulatory relationships between these TFs and genes, which may potentially lead to some experimentally testable hypotheses of gene regulations responding to influenza A virus infection.

## Conclusions

We proposed a novel pipeline for reverse-engineering of the dynamic GRN based on ODE models. We focused on the genome-wide time course gene expression data, which provides a complete view of the evolvement of the biological phenomena over a period of time rather than at a single time point. A series of advanced statistical and computational techniques are employed to efficiently reduce the dimension of the problem and to account for the correlations between measurements from the same gene. The proposed pipeline is a computationally efficient, data-driven tool bridging the experimental data, mathematical modeling and statistical analysis. More importantly, the pipeline employs a systems biology approach, allowing us to model a living system as a whole rather than a collection of individual biological entities and providing insights into the control of a part of the system while taking into account the effect on the whole system.

We implemented our proposed pipeline to build a genome-wide dynamic GRN of mouse lungs after influenza A virus infection. Our modules are composed of co-expressed genes. The functional annotations of these modules together with the between-module network topology provide valuable information about the dynamic activities in the mouse immune system. Specifically, the interconnected regulatory relationships between these modules represent the global and cooperative machinery in response to viral infection. Within each module, some gene regulators may drive the regulation control program so that genes in the module could perform certain biological functions of the immune response. These intra-module network connections suggest the regulatory relationships between these regulators and their target genes. Complete lists of the biological functions of each module and the intra-module regulatory relationships can be found in Tables S2, S3 and S4. Using the proposed data-driven methods, we delineated the hierarchical structure and functions of the genome-wide dynamic GRN and succeeded in detecting many regulatory interactions that are known to be important in mouse immune response, demonstrating the usefulness of the proposed pipeline in rewiring the temporal transcriptomic dynamics in response to viral infection. Moreover, our pipeline is a general methodology with broad applicability to time course gene expression data from a variety of biological processes, which will provide valuable insights into the systematic modeling of complicated biological systems and potentially generate data-driven hypothesis that can be further validated by biological experiments.

A global transcriptome analysis was conducted for these same gene expression data in [2] and the authors used cell-specific signature genes of the three main immune cell populations, NK, T and B cells, to study the kinetics of immune responses. We adopted completely different approaches from those in [2] to analysis the same data and our method emphasizes the time course nature of the gene expression data and takes into account the correlations between measurements from the same gene. From a data-driven point of view, we have identified co-expressed modules with expression patterns and enriched functions revealing the kinetics of immune response, which is consistent with the conclusions of [2]. Moreover, our analysis has also elucidated complex inter- and intra-module regulatory relationships of the mouse immune response to influenza infection.

In real biological systems, the regulatory relationships between two genes are not instantaneous. Model (5) can be adapted as follows to account for time delays arising from the time required to complete transcription and translation [20], [29]:
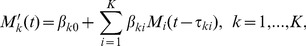
(1)where 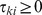 denotes time delay. To further extend model (1), we can let coefficients 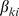 be time dependent in order to model time-varying regulatory relationships [68], [69]. We can also consider more complex nonlinear ODE models with regulatory functions being either known nonlinear functions with unknown parameters, such as the sigmoid function model [70], or some unknown functions that may be estimated using nonparametric techniques [71]. Compared to the linear ODE model, estimation of these models is computationally more intensive and usually requires more data to be measured. Further investigation is required to use these more complex models to reconstruct high-dimensional GRNs. Another important extension of the proposed pipeline is to include some prior information in the ODE network modeling. For example, if one network component is known from literature to regulate another component, this information can be incorporated in the network structure identification with no or less penalization on the corresponding regulatory coefficients in the variable selection procedure. These topics are beyond the scope of this paper and will be studied in the future research.

## Methods

### Identifying differentially expressed genes

We treat the expression profile of each gene 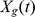 as a smooth curve of time and the time course microarray measurements are collected as discrete observations from 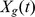 that are contaminated by noisy signals. Here we estimate 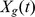 from the noisy time course data through a data-based eigen-representation: 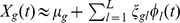, where 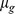 is the mean expression, 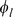 are the sequences of orthonormal eigenfunctions and 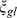 are the corresponding functional principal component scores [40]. The top 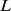 eigenfunctions are selected such that the total variation explained exceeds a pre-specified threshold (such as 90%).

In this paper, we are interested in identifying genes with significant expression changes after virus infection. So the hypothesis of testing DE genes can be written as
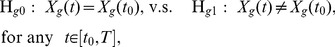
(2)where 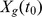 is the gene expression level at baseline. Another widely adopted hypothesis is
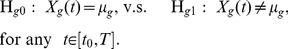
(3)


This hypothesis is used to test DE genes that have non-flat expression patterns within the time interval of interest. For hypothesis (2), we adopt the modified 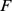-statistic in [72] to compare the goodness-of-fit of the null model to the alternative model:
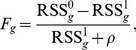
(4)where 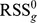 and 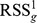 are the residual sum of squares under the null and the alternative models for the 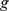-th gene, respectively. This statistic can also be viewed as the signal-to-noise ratio of each gene. For genes with a low signal level, the variance in 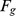 can be high because of small values of 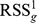. The small constant 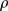 in the denominator can help stabilize the variance of 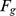 and is set as the estimated variance of the noisy signal in [72]. A permutation test is used to generate the null distribution of 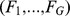 and the multiple testing adjustment method proposed by Benjamini and Hochberg [73] is applied to control the false discovery rate (FDR).

### Clustering genes into modules

For each DE gene, the replicated gene expression levels are averaged at each time point and then a gene-wise standardization procedure is applied. It has been well documented that genes with similar biological functions usually have similar expression patterns but with different expression magnitudes (fold changes) [2]. This standardization procedure can remove such magnitude differences and is commonly adopted when the goal is to group genes that are functionally related [74].

We use the K-means clustering method [75] to group co-expressed genes and propose an empirical rule to determine the number of clusters. Let 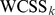 be the sum of the squared distances to the cluster centers when there are 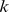 clusters. We plot the relative changes of the within-cluster sum of squares 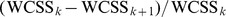 against the number of clusters 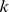 and choose the number 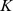 at the knee of the curve. The motivation behind this proposal is that the within-cluster variation 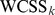 decreases as 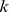 increases, and the decreasing rate should significantly slow down after 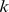 passes the optimal number of clusters [75].

### Identification of network structure

Our module-based linear ODE model for the GRN can be written as
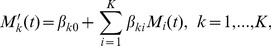
(5)where 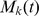 is the mean expression curve of the 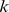-th module; 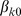 is the intercept and coefficients 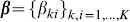 quantify the regulatory effects of the other modules, including self-regulation on the rate of expression change of the 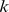-th module. The identification of network structure is equivalent to identify the nonzero coefficients 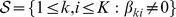.

#### Nonparametric smoothing

Within each of the 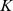 modules (super-genes), the gene expression patterns are similar, so we can treat the time course data of these genes as longitudinal measurements of the super-gene and model them using the following nonparametric mixed-effects model [76]:
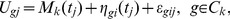
(6)where 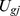 is the expression level at 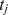 for the 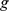-th gene, 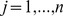, 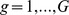; 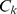 is the collection of gene indices for the 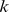-th module and 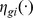 is the random-effects function that quantifies the deviation of the expression level of gene 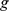 from the mean expression 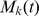. Applying mixed-effects smoothing splines [76], we can obtain the estimates of the mean expression curve 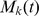 and its first order derivative 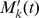 for each module. Following [32], we suggest under-smoothing these curve estimates in this step.

#### Variable selection for the linear ODE model

We plug the estimated mean expression curves and their derivatives 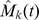 and 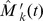 into the ODE system (5) to form a set of pseudo linear regression models. Since 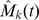 and 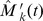 are estimated continuously as nonparametric functions, we recommend using augmented data from 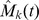 and 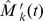 at time points 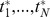 (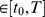), where 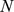 can be larger than the original sample size 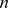. This data augmentation strategy has also been used by other investigators before [27], [77]. Denote the augmented data as 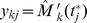 and 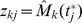, 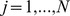. We can write the pseudo regression models as
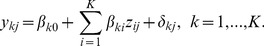
(7)


The error term 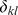 represents the aggregated estimation error of 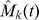 and 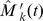 and model error due to the substitution of the differential equation variables by 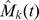 and 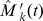. Note that these errors are dependent, and the predictors 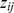 and responses 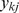 in (7) are derived from the smoothing estimates rather than directly measured data. Therefore, model (7) is not a standard regression model and this is why we refer to it as a “pseudo” linear regression model.

For each of these pseudo regression models, we apply the smoothly clipped absolute deviation (SCAD) method [78] to select nonzero 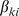
's. Without loss of generality, we assume that both the response 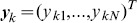 and covariates 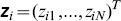, 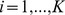 in (7) are centered, so 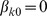. Consider the following penalized objective function
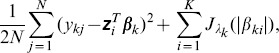
(8)where 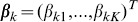 and 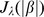 is the SCAD penalty [78]. We employ the CCCP-SCAD algorithm developed by [79] to minimize (8) and obtain the collective set of nonzero coefficients 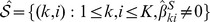, where 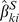 are the minimizers of (8). This set 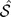 gives the structure of the ODE network model (5) and the regulatory relationships between modules in the network. A simulation study to validate the performance of the SCAD variable selection method in identifying the structure of ODE networks can be found in Materials S1.

### Refining parameter estimates

The parameter estimates obtained from the two-stage method are not efficient in terms of estimation accuracy, because of the approximation errors brought in by the estimates of the mean expression curves 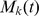 of the modules and their derivatives 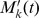. These errors could be quite large when the data are measured at a sparse grid or with large noise signals. To overcome this drawback, we propose to refine the parameter estimates for the selected ODE model using the nonlinear least squares (NLS) method [68]. The SCAD estimators 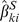 from the two-stage method can be used as the initial estimates in the NLS procedure.

### Functional enrichment analysis

Genes within the same module may have many biological functions and certain functions may be enriched in this module compared to the population of genes in an organism or a biological process. These enriched functions are the key factors to understand the role that the module plays in the whole network. We can use DAVID [41] to identify the gene ontology (GO) functional annotations and KEGG/BioCarta/Reactome to identify pathways that are enriched in each module. In this analysis, a modified Fisher's exact test is carried out for each functional term under the null hypothesis that this function is not over-represented in the module compared to the background population [41]. The statistical significance of each functional term is adjusted by the multiple testing adjustment method proposed by [73]. We collect the mouse TFs from FANTOM [58] and Uniprot [59], respectively (some of them are putative). We also integrate the curated gene regulations deposited in TRED [80] and KEGG [81], as well as the documented mouse protein-protein interactions in STRING [82]. In each module, the regulations between the TFs and targets are then coupled and analyzed.

## Supporting Information

Table S1 **A complete list of the 20 modules and their member genes.**
(XLSX)

Table S2 **The biological functions and pathways enriched in each module.** In the column Category, GOTERM_MF, GOTERM_CC, or GOTERM_BP refers to the GO ontologies for molecular function, cellular component and biological process, respectively, and KEGG_PATHWAY refers to the KEGG pathway annotations. GOTERM_MF_FAT is a GO category that filters out the broad GO terms based on a measured specificity of each term and GOTERM_MF_ALL refers to all the rest terms. _FAT and _ALL are defined similarly for the other two GO ontologies. The column Term displays the descriptions of the enriched functions or pathways. The Column Count lists the number of genes with the corresponding annotated function and “%” is the percentage of these annotated genes in the background population. The column Adj.pval lists the adjusted *p*-values by the Benjamini and Hochberg procedure and the column Gene displays the genes associated with the enriched functions or pathways.
(XLSX)

Table S3 **Mouse transcription factors (TFs) compiled from FANTOM and Uniprot.**
(XLSX)

Table S4 **The intra-module regulatory relationships in 20 modules.** The interactions from TF to Gene are collected from the databases of TRED, KEGG, and STRING.
(XLSX)

Materials S1 **Simulation study.**
(PDF)
