# Supplementary material for: Modeling Genome-Wide Dynamic Regulatory Network in Mouse Lungs with Influenza Infection Using High-Dimensional Ordinary Differential Equations
Source: PLoS One. 2014 May 6;9(5):e95276. doi: 10.1371/journal.pone.0095276 (PMC4011728; doi:10.1371/journal.pone.0095276)
Supplement: Materials S1 — Simulation study. (PDF) [file pone.0095276.s005.pdf]

Supplementary Materials of "Modeling genome-wide dynamic regulatory network in mouse lungs with influenza infection using high-dimensional ordinary differential equations"

Shuang Wu[1,†], Zhi-Ping Liu[1†], Xing Qiu[1], Hulin Wu[1,*]
1 Department of Biostatistics and Computational Biology, University of Rochester, Rochester, NY, USA
∗ E-mail: Hulin_Wu@urmc.rochester.edu
† These authors contributed equally to this work.

Simulation Study

In this section, we conduct simulation experiments to validate the performance of the key step of identifying network structure in our pipeline. We consider the following linear ODE model with 20 equations , the same dimension as the module-based network for the mouse influenza infection data,

$$\boldsymbol{M}'(t) = A\boldsymbol{M}(t), \quad t \in [0, 10], \tag{1}$$

where $\boldsymbol{M}(t) = (M_1(t), ..., M_{20}(t))^T$. The coefficient matrix A is a randomly generated sparse matrix such that model (1) is identifiable and has stable solutions. The solution to model (1) serves as the true mean expression curve of each module and the experimental data are simulated using the following model,

$$U_{ki}(t_j) = M_k(t_j) + b_{ki} + \epsilon_{kij}, k = 1, ..., 20;\ i = 1, ..., N_k;\ j = 1, ..., n, \tag{2}$$

where N_k is the number of genes in each module; n is the number of time points for each gene; b_{ki} is the random effect to quantify the deviation of gene i from the mean expression of the kth module and $b_{ki} \sim \mathcal{N}(0, \sigma_b^2)$; and the measurement error ϵ_{kij} is assumed to follow a normal distribution with mean 0 and standard deviation σ_ϵ.

We randomly generate 100 coefficient matrix A. For each simulated A, there are 88 nonzero coefficients and 2 to 6 nonzero coefficients for each differential equation. We set $\sigma_b = 0.5$ and $N_k = 100$ for each module. The standard deviation of measurement errors is chosen to be $\sigma_\epsilon = 0.05$ or 0.5. For the sample size, we consider $n = 14$, which is the same as the mouse influenza infection data and a larger sample size $n = 30$.

The accuracy of our variable selection approach is measured by sensitivity and specificity, which are defined as follows:

$$\begin{aligned} \text{sensitivity} &= \frac{\text{\# of correctly estimated edges}}{\text{\# of all edges in the true network}}, \\ \text{specificity} &= \frac{\text{\# of correctly estimated edges}}{\text{\# of all estimated edges}}. \end{aligned}$$

The averaged results of 100 simulations are displayed in Table 1. We can see that both sensitivity and specificity of the identified networks increase with the increase of sample size and decrease of measurement error variance. For the worst case (the largest variance and smallest sample size), our method can still correctly identify almost 70% of the edges. These results clearly demonstrate that the proposed method works well in identifying the structure of ODE networks.

Table 1. The average values of the total number of estimated edges, and the sensitivity and specificity of the fitted models (with standard deviations in parentheses).

n	σ	# estimated edges	Sensitivity	Specificity
14	0.5	91.30	0.6774 (0.0353)	0.6549 (0.0453)
	0.05	88.66	0.7179 (0.0281)	0.7151 (0.0468)
30	0.5	89.86	0.7219 (0.0406)	0.7101 (0.0585)
	0.05	89.32	0.7723 (0.0361)	0.7643 (0.0619)
